# The impact of intraoperative frozen section analysis on final resection margin status, recurrence, and patient outcome with oral squamous cell carcinoma

**DOI:** 10.1007/s00784-021-03964-y

**Published:** 2021-05-06

**Authors:** Katharina Nentwig, Tobias Unterhuber, Klaus-Dietrich Wolff, Lucas M. Ritschl, Markus Nieberler

**Affiliations:** Department of Oral and Maxillofacial Surgery, Technical University of Munich, School of Medicine, Klinikum rechts der Isar, Ismaninger Straße 22, 81675 Munich, Germany

**Keywords:** Head and neck, Oral squamous cell carcinoma, Intraoperative surgical margin control, Intraoperative frozen section analysis, Diagnostic value

## Abstract

**Background:**

The objective of this study was to evaluate the diagnostic value of intraoperative frozen section analysis (IFSA) of tumor bed margins in patients with oral squamous cell carcinoma (OSCC).

**Methods:**

This retrospective study includes 194 primary OSCC cases. The impact of intraoperative information by IFSA on final margin status, local recurrence, and disease-specific survival were analyzed.

**Results:**

IFSA revealed a 50% sensitivity and a 100% specificity, with a positive and negative predictive value of 100% and 89.1%, respectively. In 19 cases, margins were rated positive by IFSA and remained positive in eight cases (42.1%), despite immediate re-resection. This constellation led to higher recurrence and lower survival rates than in cases with consecutive R0 status (each *p* = 0.046). Positive margins in IFSA were associated with closer final margins (*p* = 0.022) and early recurrences (*p* = 0.050).

**Conclusions:**

Achieving instant R0 status has a crucial impact on disease recurrence and patient survival. IFSA falls short to ensure secure definite surgical margins. Thus, improved intraoperative diagnostic information on the location and extent of OSCC could support patient treatment.

**Clinical relevance:**

Considering that patient survival has not improved despite progress in surgical and adjuvant therapy, the process and outcome of IFSA was scrutinized as one part of the treatment concept.

## Introduction

The five-year survival rate of oral squamous cell carcinoma (OSCC) has not improved significantly in recent times, despite interdisciplinary efforts in specialized cancer centers and guideline-based treatment strategies [1–3]. Based on the 2012 German S3 guideline for OSCC, surgical therapy and stage-dependent adjuvant radio(chemo)therapy is still recommended as first-line therapy for affected patients [4]. The complete carcinoma resection with adequate distance to the resection margins (R0 status) is the primary objective of curative intended surgical treatment, and the impact of the final resection margin status on disease recurrence and patient outcome is well described [5–7]. Close (< 5 mm) or positive resection margins (R1 status) are regarded as a major influence on patient prognosis [8–12]. In the case of close or positive margins, re-resection of the primary tumor bed is suggested to obtain R0 status. Despite the progress of imaging techniques, there is still not enough information and experience in clinical work to clearly demarcate the boundary between healthy and diseased tissue [13, 14]. In addition, preoperative imaging information cannot be transferred routinely into the intraoperative setting without loss of information or increased technical effort. In this context, intraoperative frozen section analysis (IFSA) serves as a diagnostic tool to anticipate intraoperatively adequate resection margins in most head and neck centers, according to a survey of American Head and Neck Society members by Bulbul et al. [12]. IFSA provides immediate information on the soft tissue resection margins of the main tumor specimen or the former tumor bed and represents the standard means for the surgeon to avoid unnecessary and/or inadequate resections [4, 15].

Considering that patient survival has not improved despite progress in surgical and adjuvant therapeutic concepts, the process and outcome of IFSA should be scrutinized as one part of the whole treatment concept. We investigated if IFSA provides diagnostic validity and reliability to define the boundary of invasive carcinoma growth for adequate resection and, therefore, patients’ prognosis. IFSA was compared with final resection status (R) as well as patients’ disease recurrence and survival rate.

## Material and methods

### Ethical statement

All clinical investigations were conducted according to the principles expressed in the Declaration of Helsinki. The study was approved by the institutional ethics committee of the Technical University of Munich, School of Medicine, Klinikum rechts der Isar (455/15 s).

### Subjects

The retrospective study includes patients with histologically confirmed primary OSCC, treated between 2014 and 2017 at the Department of Oral and Maxillofacial Surgery, Technical University of Munich, School of Medicine, Klinikum rechts der Isar. Patients with the initial diagnosis of OSCC involving the tongue, floor of mouth, alveolar and buccal mucosa, or hard palate were included. Patients who met the following criteria were excluded: recurrent OSCC, primary radio(chemo)therapy, and death within the first 6 months after diagnosis of OSCC. TNM was defined by AJCC edition 7.

### Diagnostic and therapeutic procedures

**Fig. 1:**
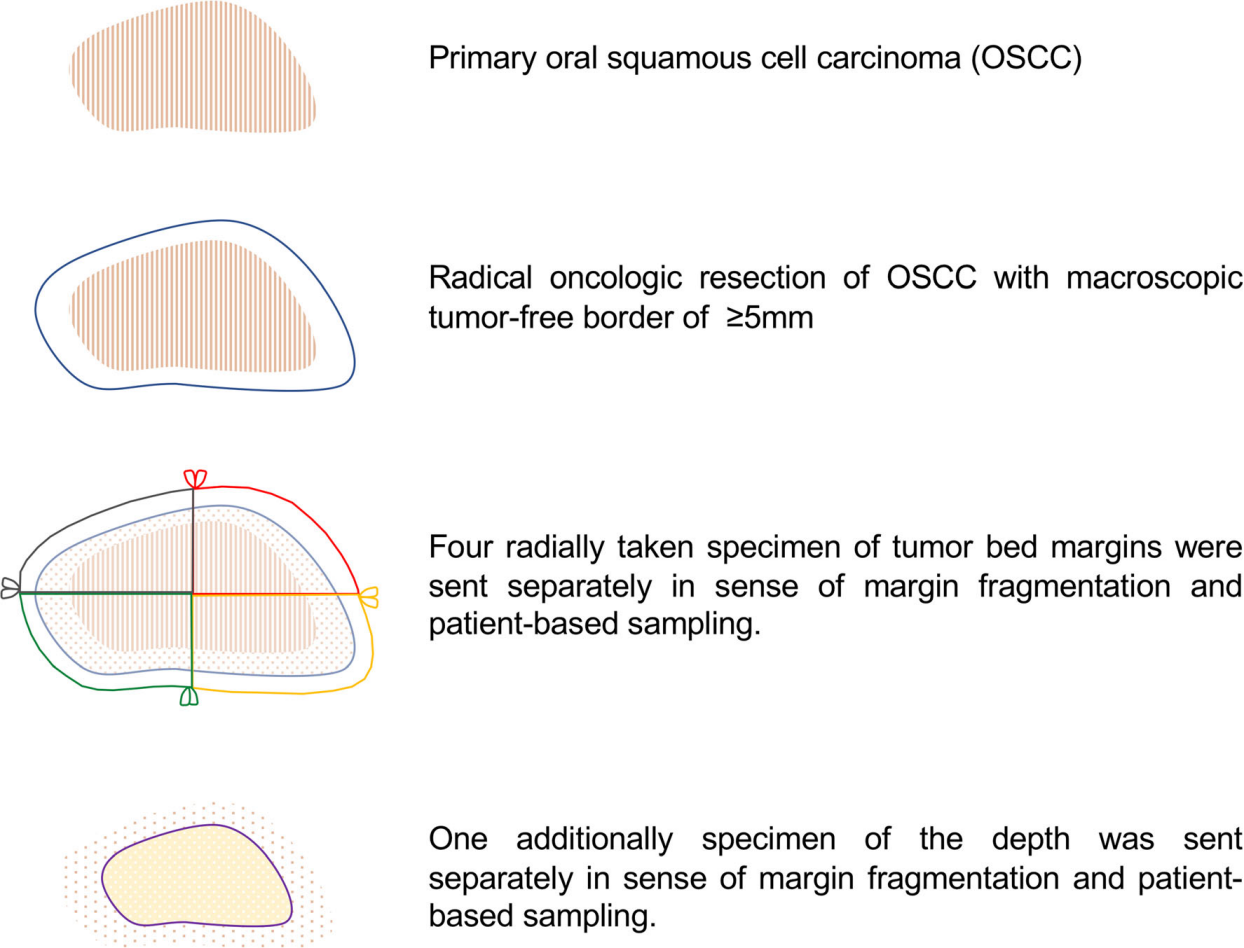
Schematic drawing of oncologic resection and systematically taken specimens for intraoperative frozen section analysis by a specialized head and neck pathologist. The specimens were taken radially (four) and of the depth (one) from tumor bed margins and sent separately in the sense of margin fragmentation and patient-based sampling. The positions of the radially taken tumor bed margins were marked clockwise and correlated with anatomical regions of the oral cavity

Surgical treatment was performed according to the individual decision of a preoperative interdisciplinary head and neck tumor board at the Department for Oral and Maxillofacial Surgery of the Technical University of Munich, Germany. Surgical treatment included radical resection of the primary tumor with a (macroscopic) tumor-free border of at least 10 mm to reduce the risk of positive margins due to mucosal shrinkage and shrinkage caused by formalin fixation [16–18]. Further, an ipsi- or bilateral neck dissection was performed in every case as an elective supraomohyoidal procedure. After the main oncologic resection, four radially taken specimens of tumor bed margins and one specimen of the depth of the tumor bed were sent separately in the sense of margin fragmentation and patient-based sampling (Fig. 1) and underwent IFSA. The positions of the radially taken tumor bed margins were marked clockwise and correlated with anatomical regions of the oral cavity to favor the final evaluation of the resection margins [19]. IFSA was performed by specialized head and neck pathologists with intraoperative consultation. In the case of positive margins (R1 status) immediate re-resection of the former tumor bed according to the corresponding, positive margins were conducted until carcinoma-free IFSA was obtained. The primary tumor was processed as a formalin-fixed main specimen in the pathological examination. The statement regarding the status and distance of the final resection margins included the margins of the main specimen and the margin distance acquired by the IFSA. Positive margins revised to negative under IFSA guidance were rated as final negative margins.

All patients were postoperatively again considered by our interdisciplinary head and neck tumor board to discuss the (final) findings of the histopathological process (IFSA and final specimen including all corresponding margins) with respect to the necessity of adjuvant therapy. In cases of (final) R1 status regarding primary tumor site, immediate re-resection of the afflicted margins was recommended if surgically possible. Adjuvant radio(chemo)therapy was recommended according to the S3 guidelines. Indications for radiotherapy include >pT2 stage, ≥R1 status, ≥pN1, and Pn1. Chemotherapy was indicated in cases with extracapsular involvement of affected cervical lymph nodes.

### Data collection and analysis

Clinical data and pathological reports were collected from the hospital SAP (system analysis and program development) database system. The data were analyzed with SPSS for Windows, version 17 (SPSS, IBM, Ehningen, Germany). We evaluated the intraoperative resection margin analysis by frozen sections, compared to the definite postoperative histological resection status, as defined by the histology of formalin-fixed and paraffin-embedded (FFPE) specimens.

### Statistical analysis

The 2-year, 4-year, and overall survival rates were calculated. The findings were compared with the chi-square (*p* < 0.05) and Fisher’s exact test (*p* < 0.05) for dichotomized variables. Univariate survival curves were calculated with the Kaplan–Meier method; distributions were compared by the log-rank test. Multivariate logistic regression analysis was performed to identify independent predictors of local recurrence and disease-specific survival (*p* < 0.05).

## Results

### Clinical and pathological data

Included patients (total *n* = 194; male = 127; female = 67; age range: 28–89) presented with a median age of 61.5 years. The predominant OSCC locations included the tongue (26.8%), the floor of the mouth (19.6%), but also overlapping, multiple locations within the oral cavity (29.9%). Most patients presented with pT1 (39.7%), followed by pT2 (27.8%), pT4 (22.7%), and pT3 (9.8%) findings. Patients revealed lymph node metastases with pN1 or higher in 38.1% of cases.

The median follow-up period was 41 months (range 36–65). The overall recurrence rate was 22.0% with an overall survival rate of 61.0%. The 2-year survival rate was 66.5% and the 4-year survival rate was 61.9%. The clinical and pathological data are summarized in Table 1.

**Table 1:** Clinical and pathological data

Parameters	Cases *n* = 194	[%]
*Gender*		
Men	127	65.5
Women	67	34.5
Age	Median, 61.5 years; range, 28–89 years	
*Localization*		
Tongue	52	26.8
Floor of mouth	38	19.6
Mandibula	26	13.4
Cheek	9	4.6
Maxilla/hard palate	4	2.1
Soft palate	7	3.6
More than one region	58	29.9
*pT stages*		
pT1	77	39.7
pT2	54	27.8
pT3	19	9.8
pT4	44	22.7
*N stages*		
pN0	120	61.9
pN1	27	13.9
pN+ (>N1)	47	24.2
*Extracapsular evasion*		
Yes	32	16.5
No	42	21.6
No invasion	120	61.9
*G status*		
G3	26	13.4
G2	138	71.1
G1	28	14.4
Not defined	2	1.1
*Intraoperative frozen section*		
Positive	19	9.8
Negative	175	90.2
*Final R-status*		
R0	167	86.1
R ≥ 1	27	13.9
*Tumor recurrence*		
Yes	43	22.2
No	151	77.8
*2-year survival rate*		
Yes	129	66.5
No	65	33.5
*4-year survival rate*		
Yes	120	61.9
No	74	38.1

### Overview of the therapeutic concept

Patients were treated on a case-by-case basis in accordance with the German S3 guideline for OSCC with surgical therapy. Adjuvant radio(chemo)therapy was performed in the case of advanced T stages (>T2), close or positive resection margins, perineural or vascular invasion, or lymph node metastasis (>N0). In summary, 101 (51.5%) patients received R(C)TX.

### Intraoperative frozen section analysis and final resection status

In 19 cases (9.8%) out of all patients, IFSA defined positive surgical margins with residual carcinoma (R1 status). Immediate re-resection was performed in all positive cases with the aim of carcinoma-free margins (R0). In 11 of the 19 cases (57.9%), intraoperative re-resection was successful in achieving final R0 status. However, despite complying with standard diagnostic and therapeutic procedures and intraoperative re-resection, a postoperative histological R1 status could not be prevented in eight out of the 19 cases (42.1%) (*p* = 0.001). Mucosal (3 cases) and deep margins (4 cases) have been equally affected (both margins in one case).

In 175 cases (90.2%), IFSA defined adequate surgical margins (R0). Out of these cases, 19 (10.9%) patients revealed residual carcinoma at the resection side in the final postoperative histological finding (R1) despite IFSA having defined carcinoma-free margins (R0). Considering this false-negative rate, IFSA revealed a 50% sensitivity and a 100% specificity, with a positive and negative predictive value of 100% and 89.1%, respectively. The accuracy was 90.2% (Table 2).

**Table 2:** Results of intraoperative margin analysis by frozen sections compared to the final histological R-status (*n* = 194)

	R-status		Sensitivity	Specificity	Accuracy
	*Negative (R0) n (%)*	*Positive (R1) n (%)*	50% (95% CI = 33.38–66.62)	100% 95% CI = 97.66–100)	90.21% (95% CI = 85.13–94)
Frozen section analysis	175 (90.2%)	19 (9.8%)			
R-status after re-resection in 19 R1-cases	11 (57.9%) (p = 0.001)	8 (42.1%)			
Final R-status	167 (86.1%)	27 (13.9%)			

Regarding the T stages, IFSA was defined positive (R1) in 4 cases with pT1 (5.3%), 6 cases with pT2 (11.1%), 6 cases with pT3 (31.6%), and in 3 cases with pT4 carcinoma (6.8%) (*p* = 0.02). Residual carcinoma in the final histological results were found in 2 patients with pT1 (2.7%), 5 patients with pT2 (9.3%), 5 patients with pT3 (26.3%), and in 14 patients with pT4 stages (31.8%) (*p* = 0.000).

### Intraoperative frozen section analysis and local disease recurrence

In 19 cases (9.8%) out of all patients, IFSA defined inadequate surgical margins with residual carcinoma (R1 status). Eleven patients (57.9%) changed to R0 status after re-resection. Among the 19 cases with initial R1 status in IFSA, five (26.3%) developed disease recurrence at the former primary tumor location (local recurrence). Of 175 patients in whom intraoperative R0 status was described by IFSA, 38 (21.7%) developed local recurrence. In summary, cases with intraoperative R1 status revealed a local recurrence rate of 26.3%, compared to 21.7% in cases with intraoperative R0 status (*p* = 0.417).

As a further step, the cases with recurrences were divided into incidences < 6 months (early) and > 6 months (late, #907) after surgical therapy. In cases with intraoperative R1 status, early recurrence occurred in exactly 50.0% of cases. In cases with intraoperative R0 status, early recurrence occurred in 26.0% (*p* = 0.050) of cases.

**Fig. 2:**
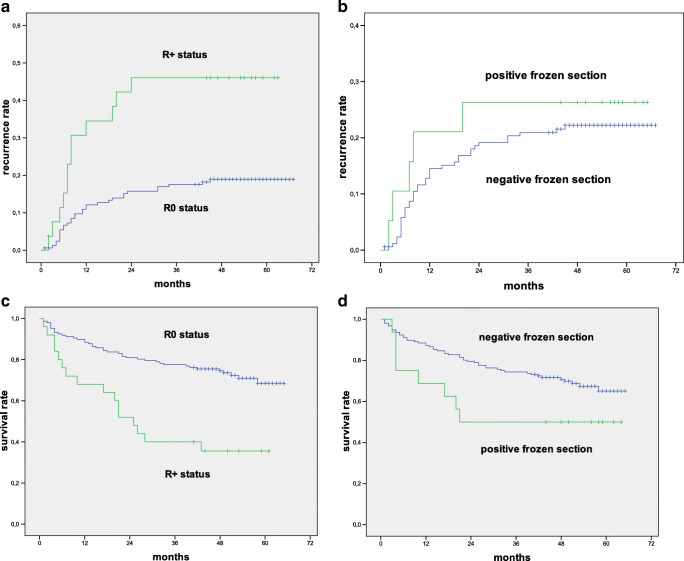
Kaplan–Meier analysis of intraoperative frozen section analysis and final R stages regarding recurrence and survival rates

In the case of local recurrence, the mean time interval for patients with a negative IFSA margin was 9 months (range 1–45), compared to 5 months (range 2–20) in cases with a positive IFSA margin (*p* = 0.278; Fig. 2).

### Final postoperative resection status and disease recurrence

In 27 (13.9%) cases, final histology confirmed the R1 status and either re-resection in a second operation or adjuvant radio(chemo)therapy was chosen. Among these cases, 12 (44.4%) developed local disease recurrence. In 167 cases, the final histology recorded R0 status. Among these cases, 31 (18.6%) developed local disease recurrence (*p* = 0.003). Thus, in cases with final R1 status, local recurrence was significantly higher (44.4%) than in cases with final R0 status (18.6%) (p = 0.003; Table 3).

**Table 3:** Impact of surgical margin control on disease recurrence and patient survival

	Frozen section analysis		*p* value	Final R-status		p value
	*Negative (R0)* *n (%)*	*Positive (R1)* *n (%)*		*Negative (R0)* *n (%)*	*Positive (R1)* *n (%)*	
*Recurrence*						
Yes	38 (21.7%)	5 (26.3%)	0.417	31 (18.6%)	12 (44.4%)	**0.003**
No	137 (78.3%)	14 (73.7%)		136 (81.4%)	15 (55.6%)	
*Survival rate*^*#*^						
2-year survival	138 (78.8%)	8 (42.1%)	**0.010**	133 (79.6%)	13 (48.1%)	**0.005**
4-year survival	127 (72.6%)	8 (42.1%)	0.155	126 (75.5%)	10 (37.5%)	**0.001**

### Resection status and distance from the primary carcinoma

Regarding the distance of the final resection margin from the primary carcinoma, we observed that in the case of a negative IFSA, the margin was 5.5 mm on average compared to a distance of 4.3 mm in the case of a positive IFSA margin (*p* = 0.022). The distance was measured by head and neck pathologists by summing the margin on the main specimen with the corresponding resections margins.

### Overall survival in relation to the intraoperative resection status

The overall survival rate in our follow-up period was 61.0%. The 2-year survival rate in cases with intraoperative R1 status was 42.1%. In contrast, cases with intraoperative R0 status revealed a 2-year survival rate of 78.8% (*p* = 0.010).

The 4-year survival rate for cases with an intraoperative R1 status was 42.1%. Cases with intraoperative R0 status revealed a survival rate of 72.9% (*p* = 0.155; Table 3).

In the case of intraoperative R1 status, the patients died after a median time of 21 months (range 3–65). Patients with intraoperative R0 status died after a median time of 48 months (range 1–65) (log rank 0.075).

### Overall survival in relation to the final resection status

Cases with final R1 status showed a significantly lower 2-year survival rate with 48.1%, compared to cases with final R0 status, which had a 2-year survival rate of 79.6% (*p* = 0.05). The 4-year survival rate for cases with final R1 status was 37.5%. Cases with final R0 status had a 4-year survival rate of 75.5% (*p* = 0.001; Table 3).

### Clinical impact of resection margin control on disease recurrence and patient outcome

In cases with positive IFSA, re-resection was performed. If re-resection was necessary, final R0 status could be achieved in 57.9% of the cases. In the case of R1 revised to R0 based on frozen section analysis, the recurrence rate was 9.0% with a 4-year survival rate of 75.0%.

Multivariate analysis revealed no significant difference between patients with a positive intraoperative resection margin that ended up in final R0 status histology and patients with a negative intraoperative resection margin with final R0 status concerning recurrence and survival (*p* = 0.141/0.134).

However, in 42.1% of the cases, re-resection was performed because of an intraoperative R1 status but failed to achieve postoperative R0 status. In these cases, the recurrence rate was 50.0% with a 4-year survival rate of 25.0% (*p* = 0.046 and 0.046).

## Discussion

Intraoperative frozen section analysis is suggested in the German treatment guidelines for OSCC [20]. But there is no recommendation or consensus whether tumor bed-driven (=patient-based) or specimen-driven sampling should be performed. Recently, specimen-driven sampling is reported to be safer in terms of gaining reliable information about the margins and is also reported to have lower local recurrence rates [14, 19]. Bulbul et al. (2020) revealed in a survey of American Head and Neck Society members that 55% apply specimen-driven and 45% tumor bed-driven intraoperative sampling. In the case of close or positive resection margins, an immediate intraoperative re-resection should be performed aiming for clear surgical margins. The definition of free resection margin is still controversially debated but is commonly understood to be a distance of at least 5 mm from the infiltrative boundary [12]. However, the definitions of safety margins have varied in literature between 2 and 10 mm [16, 21]. Inadequate distance to the resection margin increases the recurrence rate [8, 22]. In this context, it is still controversially discussed, if findings of IFSA have the diagnostic value to support treatment regimens and consequently improve patient outcome.

A crucial point of our study is that if R0 resection was missed initially, adequate intraoperative re-resection failed in 42.1% of cases (*p* = 0.001) resulting in R1 status in the final histological finding. Nevertheless, IFSA with immediate re-resection could revise an intraoperative R1 to final R0 status, but this curative goal could only be achieved in 57.9% of the cases. However, after re-resection the widths of the final free resection margins were significantly lower in the case of initial close or inadequate resection margins (*p* = 0.022). This outcome resulted despite compliance with the standard diagnostic and therapeutic procedures of immediate re-resection.

Nineteen cases were negative in IFSA that turned out to be positive according to the final histological assessment. Overall, IFSA revealed a sensitivity of 50.0% and a specificity of 100%. These findings underline the importance of gaining precise intraoperative information on resection margin status. Failures may be caused by missing affiliation of the margins regarding frozen section analysis and main specimen and, therefore, result in inaccuracies in the pathology analysis. Especially OSCC with advanced T stages were associated with higher rates of residual carcinoma in IFSA as well as in final histological results (*p* = 0.002/0.000). In a further investigation, patients with initial positive frozen section who were re-resected were significantly associated with worse local cancer control and had a poorer disease-specific survival rate, compared to cases with initial negative margins [23]. Further, a better prognostic outcome was demonstrated if revision was conducted due to initial positive resection analysis [24]. However, if the resection margin status is unclear within the context of complicated anatomical conditions after the resection, revisions are meant to be extended in order to avoid final R1 status.

DiNardo et al. estimated that only 5% of patients benefited from immediate intraoperative margin revision. In their investigation 40% of patients with positive final margins and 100% of patients with close margins were not detected by intraoperative frozen section analysis. It was further postulated that positive or close resection margin status is the strongest predictor of local tumor recurrence [25]. Especially patients who were re-resected until final R0 status, a higher rate of relapses was observed. Our study shows that local disease recurrence was higher in cases with positive IFSA, although this was not statistically significant. As re-resection of initial positive or close margins turned into definite R0 status, we did not observe statistical differences compared to cases with initial negative IFSA. Our result contradicts the findings of other recent studies that treat formally revised R0 status as positive margins and assign adjuvant therapy [12, 26, 27]. In our analysis, IFSA led to worthy prognostic information for adequate resection provided by clear affiliations of the specimen as well as close cooperation of the surgeons with pathologists. This underlines the importance of accurate intraoperative resection control and communication between the two disciplines.

Further, the periods until local recurrence were similar in both groups. Regarding patient survival, however, we demonstrated lower rates after a 2-year and a 4-year follow-up period if close or positive resection margins were diagnosed and revision was conducted (*p* < 0.05). Patients with positive IFSA died earlier, compared with negative IFSA results. Some authors confirmed a lower survival rate in cases with positive frozen section margins where re-resection was performed [23, 26–28]. Interestingly, a clear benefit of IFSA regarding local recurrence and overall survival has not been proven, yet. This again highlights the importance of the initial demarcation of carcinoma tissue and subsequent adequate resection on patient survival. Due to the lack of preoperative and intraoperative diagnostic means for a specific demarcation of carcinoma tissue, real-time imaging specific for invasive carcinomas would serve to close this diagnostic gap.

Another disadvantage of IFSA seems to be the limited metric assessment of resection margins. Several studies already criticized mistakes as well as loss of information in the processing and transmission of the revised specimen [29, 30]. One reason for this could be the lack of close communication between the surgeon and the pathologist. Another source of mistakes may be the topographical affiliation of the obtained specimens in relation to the tumor bed.

We cannot declare IFSA as an accurate predictor of postoperative management and prognosis of patients. However, our findings emphasize that IFSA provides information for the surgeon to avoid unnecessary extended and uncontrolled resections, but it will not improve the rate of initial R0 margins.

The discussed points underline the importance of improving intraoperative tumor visualization to further improve immediate R0 status. Hinni et al. described the need for additional information and the upcoming possibilities with new methods, including molecular margins (Hinni, et al., 2013). Nieberler et al. described the use of arginylglycylaspartic acid (RGD) containing peptides that target integrin αvβ6 as a potential approach for a fluorescence-assisted intraoperative cytological assessment (Nieberler, et al., 2018). This new approach was diagnostic with a sensitivity and specificity of 100% and 98.3% and was associated with a positive predictive value of 92% and negative predictive value of 100%, compared with the cytological findings. More current practices to overcome the shortcomings of IFSA include nonfluorescent dyes, fluorescent dyes, autofluorescence imaging, narrow-band imaging, optical coherence tomography, confocal microscopy, high-resolution, microendoscopy, and spectroscopy [14]. Another feasible approach is the use of intraoperative near-infrared fluorescence (NIRF) imaging of invasive carcinomas in real time [31]. Information of the borders of invasive carcinoma growth might be an improvement to better define the boundary of healthy and diseased tissue. This will inevitably facilitate the demarcation of clear resection margins. Additionally, augmented reality-assisted tumor resection might be one upcoming tool to facilitate resections of large tumors or in complex anatomic regions. Altogether, the application of the new technologies and strategies all pursue the essential goal of initial complete tumor resection, which can be considered a key factor in further improving overall survival rates. Further studies will have to show which technique for intraoperative margin control remains a worthy means for surgeons to directly control one of the major risk factors for patient outcome.

## Conclusions

The data revealed that R0 status is a crucial factor that influences disease recurrence and patient survival. Consequently, intraoperative information in the sense of real-time visualization to achieve final R0 status is highly valuable. The current intraoperative diagnostic standard by IFSA enables re-resection in the case of R1 status to improve recurrence rates and patient survival.

However, the diagnostic value of intraoperative margin control by IFSA and its therapeutic consequences is limited, because inadequate surgical margins may not be corrected, despite immediate re-resection with the curative intention to achieve R0 status. Consequently, a higher rate of cases with final R1 status was revealed in the case of inadequate initial resection. It is therefore necessary to improve intraoperative means to gain information on the location and extent of invasive carcinomas before resection.
